# Transmission dynamics and elimination potential of zoonotic tuberculosis in morocco

**DOI:** 10.1371/journal.pntd.0005214

**Published:** 2017-02-02

**Authors:** Mahamat Fayiz Abakar, Hind Yahyaoui Azami, Philipp Justus Bless, Lisa Crump, Petra Lohmann, Mirjam Laager, Nakul Chitnis, Jakob Zinsstag

**Affiliations:** 1 Institut de Recherches en Elevage pour le Développement, N’Djaména, Chad; 2 Swiss Tropical and Public Health Institute, Basel, Switzerland; 3 University of Basel, Basel, Switzerland; 4 Institut Agronomique et Vétérinaire Hassan II, Rabat, Morocco; University of Iowa, UNITED STATES

## Abstract

Bovine tuberculosis (BTB) is an endemic zoonosis in Morocco caused by *Mycobacterium bovis*, which infects many domestic animals and is transmitted to humans through consumption of raw milk or from contact with infected animals. The prevalence of BTB in Moroccan cattle is estimated at 18%, and 33% at the individual and the herd level respectively, but the human *M*. *bovis* burden needs further clarification. The current control strategy based on test and slaughter should be improved through local context adaptation taking into account a suitable compensation in order to reduce BTB prevalence in Morocco and decrease the disease burden in humans and animals. We established a simple compartmental deterministic mathematical model for BTB transmission in cattle and humans to provide a general understanding of BTB, in particular regarding transmission to humans. Differential equations were used to model the different pathways between the compartments for cattle and humans. Scenarios of test and slaughter were simulated to determine the effects of varying the proportion of tested animals (p) on the time to elimination of BTB (individual animal prevalence of less than one in a thousand) in cattle and humans. The time to freedom from disease ranged from 75 years for p = 20% to 12 years for p = 100%. For p > 60% the time to elimination was less than 20 years. The cumulated cost was largely stable: for p values higher than 40%, cost ranged from 1.47 to 1.60 billion euros with a time frame of 12 to 32 years to reach freedom from disease. The model simulations also suggest that using a 2mm cut off instead of a 4mm cut off in the Single Intradermal Comparative Cervical Tuberculin skin test (SICCT) would result in cheaper and quicker elimination programs. This analysis informs Moroccan bovine tuberculosis control policy regarding time frame, range of cost and levels of intervention. However, further research is needed to clarify the national human-bovine tuberculosis ratio in Morocco.

## Author Summary

Tuberculosis is a disease of humans and animals which mainly affects the lungs but can also manifest in other organs. A variety of tuberculosis bacteria cause the disease and are usually transmitted through air, i.e. inhalation of aerosols. Bovine tuberculosis (BTB) occurs predominantly among domestic cattle, although wild animals are an important reservoir for transmission. Humans are usually infected with BTB through contaminated dairy products or close contact with cattle. While BTB has been eliminated in cattle and human populations of most high-income countries, it is still a major health threat in low- and middle-income countries. In Morocco, the disease frequently occurs in cattle and poses a health risk for humans. An effective intervention to reduce BTB among domestic cattle and reduce the risk to humans is slaughter of cattle which test positive for the disease. We simulated BTB in the Moroccan cattle and human populations using a disease transmission model. We assessed effects of test and slaughter in regard to elimination of disease in cattle and humans and estimated the associated costs with the model. The time to elimination of disease depended on the number of cattle tested. Our model suggests that the disease might be eliminated in cattle within 32 years if 40% of Moroccan cattle are tested annually and infected individuals are slaughtered or within 13 years if at least 90% of the cattle population are tested. The estimated total costs for the time periods until elimination ranged from 1.55 billion euros for testing and slaughter at 50% to 1.48 billion euros at 90%. These results can be used as a guide for planning BTB control policy in Morocco with regard to time frames and associated costs.

## Introduction

Bovine tuberculosis (BTB) is a zoonotic bacterial infection caused by *Mycobacterium bovis*. It belongs to a group of well-known and newer mycobacteria, together with *Mycobacterium tuberculosis*, all of which derive from a common ancestor forming the *Mycobacterium tuberculosis* complex (MTBC) [1–4]. *M*. *bovis* is capable of infecting a broad range of hosts, including ruminants (predominantly domestic cattle), humans and other primates [5–10]. The wide host range makes BTB highly relevant to conservation projects and difficult to eliminate where wildlife reservoirs are involved, for instance, badgers (*Meles meles*) in the United Kingdom [11, 12].

Bovine tuberculosis infection in cattle is a chronic disease which first affects the lymph nodes and from weeks to decades later can affect lungs. The disease can also be manifested in other organs such as, mammary tissue, and the gastrointestinal or urinary tract. Since transmission between cattle occurs predominantly through aerosol inhalation [13–17], the transmission rate is increased by risk factors such as high herd density and intensive breeding [18]. Pseudo-vertical transmission from cows to suckling calves through infected milk has been described [19]. Factors like a long survival period for the microbes in manure and soil and close contact between animals, for example around water sources, also contribute to an increased risk of infection [20, 21]. In humans, contaminated dairy products are considered to be the main source of BTB infection, usually resulting in extra-pulmonary infection such as lymphadenitis [22–24]. These patients are missed by thoracic radiographic screening and the resulting diagnostic cascade [25]. Aerosol cattle-to-human transmission can occur during close contact with infected animals, posing an occupational risk, especially for pastoralists and farmers [2, 26]. Infection risks linked to local cultural practices, for instance consumption of fresh blood, are reviewed by Daborn [27]. There is evidence that human patients can transmit BTB to animals, and human to human transmission occurs [28, 29].

There is a bottleneck in detecting human BTB cases because the routine diagnostic protocols were developed for patients with pulmonary tuberculosis, as caused by *M*. *tuberculosis*. Tuberculosis (TB) and BTB cannot be distinguished on the basis of clinical symptoms, radiography or histopathology [30]. Glycerol-containing Löwenstein-Jensen medium, the long-time gold standard for TB culture, inhibits the growth of *M*. *bovis*, thereby increasing the number of undetected cases [31]. New molecular diagnostic tools, for example spoligotyping, and even whole genome sequencing have been developed for *M*. *bovis* detection [32, 33]. Although they require enhanced laboratory infrastructure and personnel training which are not currently available in some developing countries, these new techniques offer promise for epidemiological research, control and adequate treatment, particularly since *M*. *bovis* is resistant to pyrazinamide, one of the first-line antibiotics for TB treatment [34].

Morocco is transitioning from extensive pastoralist livestock and dairy production to more intensified production due to increasing demands for dietary protein by a growing human population [35]. The shift in agricultural practice and increased use of high-producing Holstein cattle in place of local breeds may have an impact on BTB epidemiology and contribute to a higher prevalence [36]. The official national control program in Morocco is currently based on a test and slaughter scheme. However, large-scale application remains challenging because testing is not mandatory, and the proposed compensation, ranging from 470 euros for local breeds to 980 euros for improved breeds, is considered lower than market value.

In Morocco, BTB is an endemic zoonosis in livestock. Even though the predominant livestock species in Morocco are sheep and goats, cattle remain of major importance. The most recent national survey, conducted in 2004, showed an individual cattle prevalence of 18% and a herd prevalence of 33% [37]. This prevalence remained similar in the individual level (20%), while the herd prevalence increased (58%) in a 2012 pilot study of 1,200 cattle using the tuberculin skin test [38]. Since 2000, the health risk of tuberculosis in Morocco has been addressed through a national TB program funded by the Ministry of Health in collaboration with the World Health Organization (WHO). In 2014, TB caused 2’800 deaths in Morocco [39], and human tuberculosis had a relatively high incidence, with 36’000 new cases (106 cases per 100,000 inhabitants) [40]. These data do not appear to differentiate between *M*. *tuberculosis* and *M*. *bovis* infection. A recent meta-analysis estimated the median proportion of human BTB among all TB cases in 13 African countries at 2.8%, with a range of 0–37.7% [41]. National prevalence data from a range of countries worldwide were summarized in a 2014 review; in Mexico up to 13% of all TB cases are reportedly due to BTB, while in the United States it is only 1.4% [42]. In Morocco, Bendadda et al. reported *M*. *bovis* prevalence of 17.8% among drug resistant TB isolates from 200 human sputum samples [43].

In the early 20^th^ century, the prevalence in German cattle herds was 90% [44], with 25–80% in other European states and only 2–10% in the US [45]. In most industrialized countries, the health risk and economic loss from *M*. *bovis* were considerably reduced or eliminated through strict test-and-slaughter and meat inspection protocols for cattle, along with the implementation of milk pasteurization and financial compensation of farmers [45]. Using a similar control strategy, Switzerland eradicated BTB in 1960 [46]. In most developing countries where the disease is endemic, such measures are not feasible due to financial constraints, particularly for farmer compensation, and inadequate veterinary services [47]. Alternatives for BTB endemic countries to reduce the health and economic risk related to the disease must be sought.

Although cost estimation is difficult due to immense local variability in production parameters and prices [48], the global economic loss due to BTB is thought to be about 3 billion USD annually [49]. In cattle, the disease has a significant economic impact, through an increased death rate and decreased milk and meat production, draft power and fertility [50]. Modelling approaches have been used, mainly for developing countries, to estimate different parameters and factors related to BTB. Previous publications on the economics of BTB focus mainly on the cost of disease and control efforts. Analyses of the profitability of control efforts are very scarce [51]. A study on the economics of BTB in Africa showed higher losses in intensive dairy systems in peri-urban areas of Ethiopia when compared to extensive pastoral production systems in rural areas but did not include the cost to public health [48]. Zinsstag et al presented a simplified framework for a model of animal to human transmission in which transmission between cattle and from cattle to humans is considered [51]. This model allows the simulation of different scenarios over 5–10 years, with and without intervention, where the measurable outcome is prevalence in humans and in cattle. The economic analysis further delineates broader issues such as inter-sectorial contributions from agricultural and public health or private households affected by BTB.

Interventions to reduce health and economic risks, such as those related to BTB, are non-linear processes. Although, statistical analyses of data have been used for many years to analyze different types of health interventions, mathematical modeling represents an alternative approach which provides a broader understanding, especially regarding disease transmission to humans.

Several mathematical BTB models have been developed to study the transmission dynamics and to assess the effectiveness of control measures mostly for wildlife (badgers and possums) but also for cattle [52]. In Italy, a compartmental stochastic model of within and between-farm BTB dynamics in cattle was developed using a monte-Carlo simulation of BTB epidemics following the random introduction of infected individuals in the network. Consequently, slaughterhouse inspection has been found to be the most effective surveillance component [53]. The same methodology was used in Great Britain in order to study BTB transmission dynamics and to assess the currently used control measures [54]. A model composed of two sub-models for buffalo and cattle populations in South Africa showed that BTB infection is only sustained in cattle and buffaloes when all transmission routes are involved [55]. In France, a compartmental stochastic model was used to assess within-herd spread of BTB operating in discrete time in order to design, calibrate and validate a model of spread of *M*. *bovis* within a cattle herd. Various herd management practices as well as control programs were parameterized into the model. Therefore, the median effective reproductive ratio was estimated to be 2.2 and 1.7 respectively in beef and dairy herds [56]. This paper presents an ordinary differential equation (ODE) mathematical model of BTB transmission from cattle to humans in order to estimate the disease cost and simulate potential interventions in Moroccan cattle.

## Materials and methods

### Epidemiological data collection

Annual data on cattle numbers are estimated using data routinely collected by the Ministry of Agriculture and reported to veterinary services. Our model considered these data from 1995 to 2013. In the transmission model, the cattle population was not stratified by age and sex. We used a tuberculin prevalence of 18% for cattle, as reported in the most recent national survey (2003) [33]. A similar prevalence was noted in a smaller study performed in 2012 in Sidi Kacem, Morocco [38].

### Model

**Fig 1:**
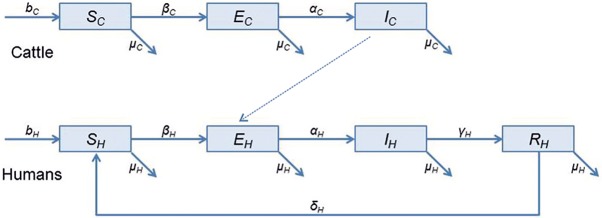
Schematic diagram of the BTB cattle-human transmission model for Morocco.

A schematic diagram of the model is depicted in Fig 1 and the variables and parameters are described in Tables 1 and 2, respectively.

**Table 1:** Description of the variables of the BTB model for Morocco.

Variable	Interpretation
*S*_*C*_	Population of susceptible cattle
*E*_*C*_	Population of exposed cattle with latent BTB
*I*_*C*_	Population of infected cattle with active BTB
*S*_*H*_	Population of susceptible humans
*E*_*H*_	Population of exposed humans with latent BTB
*I*_*H*_	Population of infected humans with active BTB
*R*_*H*_	Population of humans temporarily immune to BTB

**Table 2:** Description of the parameters of the BTB model for Morocco.

Parameter	Interpretation
*b*_*C*_	Birth rate of cattle
*β*_*C*_	Cattle to cattle transmission rate
*μ*_*C*_	Mortality rate of cattle
*α*_*C*_	Inverse of cattle incubation period
*b*_*H*_	Birth rate of humans
*β*_*H*_	Cattle to human transmission rate
*μ*_*H*_	Natural mortality rate of humans
*α*_*H*_	Inverse of human incubation period
*γ*_*H*_	Treatment success rate of humans
*δ*_*H*_	Loss of immunity in humans

The cattle population is divided into three mutually exclusive compartments consisting of susceptible cattle (*S*_*C*_), exposed cattle with latent BTB which are positive to the tuberculin test, without showing gross visible lesions (*E*_*C*_) and infected cattle with active BTB showing tuberculosis lesions (*I*_*C*_). Those parameters were estimated based on Ngandolo et al 2009 [57]. The total cattle population (*N*_*C*_) at time t is:

$$N_{C}\left(t\right)\;=\;S_{C}\left(t\right)\;+\;E_{C}\left(t\right)\;+\;I_{C}\left(t\right)$$ (1)

The human population consists of four mutually exclusive compartments: susceptible humans (*S*_*H*_), exposed humans with latent BTB reacting to the Mantoux test (*E*_*H*_), infected humans with active BTB (*I*_*H*_) and humans recovered from BTB with temporary immunity (*R*_*H*_). The total human population (*N*_*H*_) at time *t* is:

$$N_{H}\left(t\right)\;=\;S_{H}\left(t\right)\;+\;E_{H}\left(t\right)\;+\;I_{H}\left(t\right)\;+\;R_{H}\left(t\right)$$ (2)

The susceptible cattle population (*S*_*C*_(*t*)) increases through birth (at a rate *b*_*C*_) and decreases through exposure to BTB (at a rate *β*_*C*_) and mortality (at a rate *μ*_*C*_). Exposure to BTB is assumed to be frequency dependent. According to Bernues [58], the rate *b*_*C*_ decreases by 5% for exposed cattle with latent BTB (*E*_*C*_(*t*)) and for infected cattle with active BTB (*I*_*C*_(*t*)), such that:

$$\frac{dS_{C}}{dt}=b_{C}S_{C}+\left(0.95\times b_{C}\left(E_{C}+I_{C}\right)\right)-\beta_{C}\frac{I_{C}S_{C}}{N_{C}}-\mu_{C}S_{C}-\left(1-sp\right)pSc$$ (3)

The population of exposed cattle with latent BTB (*E*_*C*_(*t*)) is generated through infection of susceptible cattle with BTB (at a rate *β*_*C*_) and decreased through the development of active BTB (at a rate *α*_*C*_) and through mortality (at a rate *μ*_*C*_). Consequently:

$$\frac{dE_{C}}{dt}=\beta_{C}\frac{I_{C}S_{C}}{N_{C}}-\alpha_{C}E_{C}-\mu_{C}E_{C}-se*pEc$$ (4)

Similarly, the infected cattle population with active BTB (*I*_*C*_(*t*)) is generated through the development of active BTB among exposed cattle with latent BTB (at a rate *α*_*C*_) and decreased through mortality (at a rate *μ*_*C*_):

$$\frac{dI_{C}}{dt}=\alpha_{C}E_{C}-\mu_{C}I_{C}-se*pIc$$ (5)

For simplicity, no additional mortality rate due to BTB is assumed for the cattle and human populations in the model.

The susceptible human population (*S*_*H*_(*t*)) increases through birth (at a rate *b*_*H*_) and through recovered humans becoming susceptible again (at a rate *δ*_*C*_). The population decreases through exposure to BTB from cattle with active BTB (at a rate *β*_*H*_) and through natural mortality (at a rate *μ*_*H*_). For the susceptible human population, the exposure (both direct (aerosol) and indirect (milk) transmission) to BTB from cattle with active BTB is assumed to be frequency dependent and proportional to the number of infected cattle (*I*_*C*_):

$$\frac{dS_{H}}{dt}=b_{H}S_{H}-\beta_{H}\frac{I_{C}S_{H}}{N_{C}}-\mu_{H}S_{H}$$ (6)

Human to human transmission is assumed to be negligible. The population of exposed humans with latent BTB (*E*_*H*_(*t*)) is generated through infection of susceptible humans with BTB (at a rate *β*_*H*_) and decreased through the development of active BTB (at a rate *α*_*H*_) and through natural mortality (at a rate *μ*_*H*_):

$$\frac{dE_{H}}{dt}=\beta_{C}\frac{I_{C}S_{H}}{N_{C}}-\alpha_{H}E_{H}-\mu_{H}E_{H}$$ (7)

The infected human population with active BTB (*I*_*H*_(*t*)) is generated by the development of active BTB among exposed humans with latent BTB (at a rate *α*_*H*_) and decreased by recovery of humans with active BTB due to treatment (at a rate *γ*_*H*_) and by natural mortality (at a rate *μ*_*H*_):

$$\frac{dI_{H}}{dt}=\alpha_{H}E_{H}-\gamma_{H}I_{H}-\mu_{H}I_{H}$$ (8)

The population of humans recovered from BTB and temporarily immune due to treatment is generated through the recovery of humans with active BTB (at a rate *γ*_*H*_) and decreased through humans becoming susceptible to BTB again after the end of the prophylactic period of the drugs (at a rate *δ*_*H*_) and through natural mortality (at a rate *μ*_*H*_), so that:

$$\frac{dR_{H}}{dt}=\gamma_{H}I_{H}-\delta_{H}R_{H}-\mu_{H}R_{H}$$ (9)

### Values for variables and parameters of the model

#### Variable starting values

The most recent estimate (2013) for the cattle population in Morocco is 3,173,000 (*N*_*C*_), of which 18% [95% CI: 16.5%-20.3%] are tuberculin skin test positive. The initial values of the three compartments *I*_*C*_, *E*_*C*_ and *S*_*C*_ were calculated such that the pre-intervention endemic equilibrium of the model equals the prevalence of 18% (S1 Supporting information). This yield:

$$I_{C}=\frac{\alpha_{c}\varphi_{C}}{\alpha_{C}+b_{C}-\left(b_{C}-r_{b}b_{C}\right)\varphi }N_{C}$$ (10)

$$E_{C}=\left(\varphi_{C}-\frac{I_{C}}{N_{C}}\right)N_{C}$$ (11)

$$S_{C}=N_{C}-E_{C}-I_{C}$$ (12)

The human population of Morocco in 2013 is estimated to be 33,008,150 individuals [59]. The estimated number of people with active TB is 43,000 (prevalent cases) [40]. In Africa, a median of 2.8% of all human TB cases are caused by BTB [41]. Between 5 and 10% (mean 7.5%) of TB-exposed people will develop active TB during their lifetime [60]. The starting values of the variables are listed in Table 3. Based on these estimates the starting values of *I*_*H*_, *E*_*H*_, *R*_*H*_ *and S*_*H*_ are calculated as:

$$I_{H}=\frac{\alpha_{H}\varphi_{H}}{\alpha_{H}+\delta_{H}+b_{H}}N_{H}$$ (13)

$$E_{H}=\left(\varphi -\frac{I_{H}}{N_{H}}\right)N_{H}$$ (14)

$$S_{H}=N_{H}-E_{H}-I_{H}$$ (15)

$$R_{H}=\;0$$ (16)

**Table 3:** Initial values for the BTB cattle-human transmission model (S3 Supporting information).

Variable	Starting value
*S*_*C*_	2,601,860
*E*_*C*_	79,610
*I*_*C*_	491,529
*S*_*H*_	33,006,946
*E*_*H*_	784
*I*_*H*_	420
*R*_*H*_	0

#### Parameter values

The average lifespan of the Moroccan cattle is 6 years which yields a death rate of *μ*_*C*_ = 0.167. Form data on cattle populations (S3 Supporting information) using least squares the birth rate was estimated as *b*_*C*_ = 0.177.

From the UN World Population Prospects we calculated the birth rate *b*_*H*_ and death rate *μ*_*H*_ in humans [59]. Although BTB bacteria are not completely eliminated from treated humans [61, 62] it is nevertheless assumed, for the sake of simplicity, that all successfully treated humans are recovered and become susceptible again within a period of 6 months.

The cattle to cattle transmission rate, *β*_*C*,_ and the cattle to human transmission rate, *β*_*H*_, were estimated from the pre-intervention endemic equilibrium (S2 Supporting information). The model was implemented using MATLAB (MathWorks, Natick, MA). Table 4 shows the baseline values of the parameters with the respective source.

**Table 4:** Parameters of the BTB cattle-human transmission model assuming a stable prevalence (endemic stability).

Parameter	Baseline value		Source
*b*_*C*_(year^-1^)	0.177	[0.121–0.274]	S3 Supporting information
*β*_*C*_(year^-1^)	0.249	[10.244–0.255]	Estimated from the endemic prevalence in cattle
*μ*_*C*_ (year^-1^)	0.167	[0.111–0.333]	[63]
*α*_*C*_ (year^-1^)	1.083	[0.5–1.667]	[1]
se	0.438	[0.057–0.819]	[57]
sp	0.894	[0.842–0.946]	[57]
*b*_*H*_ (year^-1^)	0.0229		[59]
*β*_*H*_(year^-1^)	0.00015		Estimated from the endemic prevalence in humans
*μ*_*H*_ (year^-1^)	0.0063		[59]
*α*_*H*_ (year^-1^)	1.083		
*δ*_*H*_ (year^-1^)	2		[64]

The reproductive number of the transmission between cattle R_0_ was computed as:

$$R_{0}=\frac{\alpha *\beta }{\left(\alpha +\mu \right)\mu }$$ (17)

### Sensitivity analysis of model

A sensitivity analysis of the model recalculated the change of prevalence if individual parameters varied from baseline over 30 years.

### Simulation and cost of interventions

Although Morocco has a test and slaughter policy for the control of BTB, it is currently not effectively implemented. The BTB transmission model was used to estimate the effect of the proportion of tested and slaughtered tuberculin positive animals on the duration to reach freedom from disease, achieving an individual animal prevalence of less than one in a thousand tested animals (<1/1000) according to the standards of the World Organization for Animal Health (OIE) [65]. The proportion of tested and slaughtered animals was simulated by removing 10–100% of the exposed (E_c_) and infectious cattle (I_c_) per year from the herd. The control reproductive number **R**_**c**_ including the test and slaughter intervention as proportion p with a test of sensitivity se was computed as:

$$R_{c}=\frac{\alpha *\beta }{\left(\alpha +\mu +se*p\right)\left(\mu +se*p\right)}$$ (18)

The associated costs were estimated in a summaric way, assuming an incremental cost of comparative intradermal or interferon gamma (BOVIGAM) testing of 3 euros per animal. The cost of compensation at 80% of the market value varies from 470 euros for local breeds to 970 euros for improved breeds [66]. For the current estimation of the cost of BTB elimination in Morocco, we used a single value of 500 euros of compensation per slaughtered animal. Models run with and without interventions were simulated using data from 2013 onwards.

The OIE recommended cut-off for SICCT interpretation is 4 mm, however, many studies showed that a severe cut-off of 2mm increased the sensitivity of the test [67, 68], without affecting the specificity compared to the recommended cut-off [69]. Consequently, we decided to consider both 2mm and 4mm cut-offs in the model, and to compare the respective results. The present model considered both options of SICTT cut-off at 2mm and 4mm.

## Results

### Model properties

**Fig 2:**
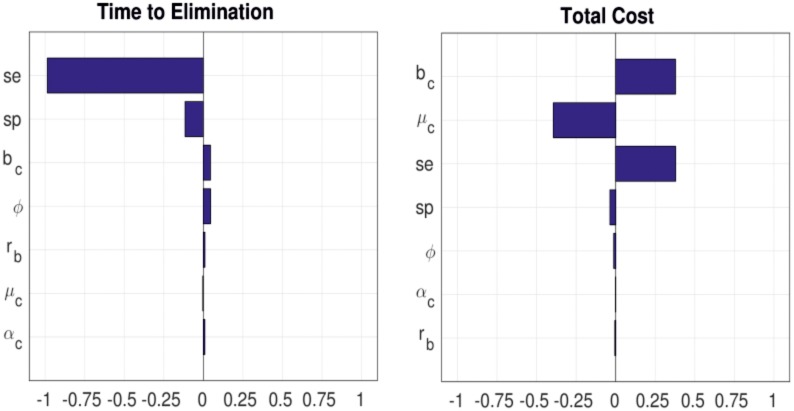
Partial rank correlation coefficients (PRCC) sensitivity analysis of time to elimination (left) and total cost (right) on parameter values.

The reproductive ratio of the cattle to cattle transmission of bovine tuberculosis without intervention was 1.325. For the total cost, the birth rate of cattle b_c_ was the most sensitive parameter influencing the dynamics of BTB transmission (Fig 2). High birth rate values lead to an increased cattle population yielding higher costs for elimination. For the time to elimination, the sensitivity of the test was the most sensitive parameter. Low test sensitivity (i.e. with cut-off at 4mm) leads to low detection of infected animals and therefore less culling of infectious cattle, which leads to a longer time to elimination.

**Fig 3:**
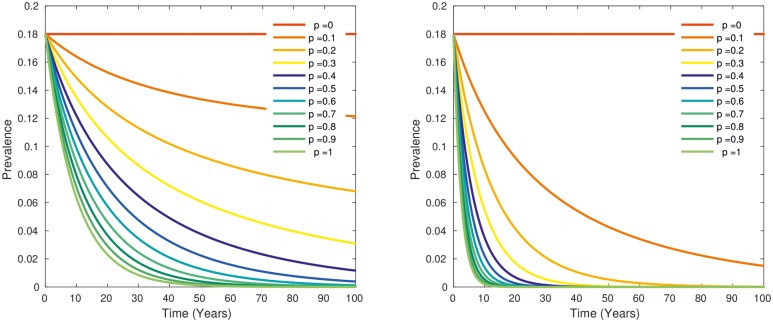
Prevalence of tuberculin positive cattle depending on the proportion of test and slaughter between 0 and 1 (in steps of 0.1) with sensitivity and specificity of the 4mm cutoff test (left) and the 2mm cuttoff test (right).

**Fig 4:**
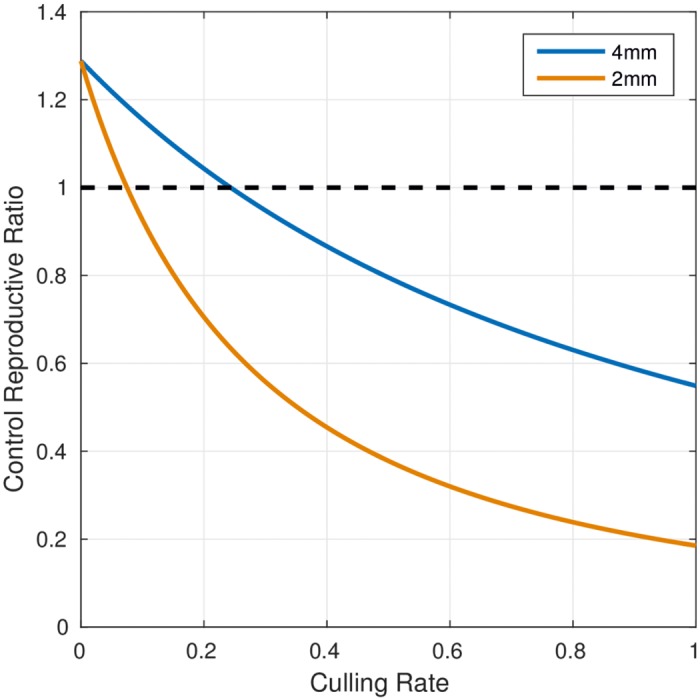
Relationship between reproductive number and proportion of test and slaughter for the 4mm cutoff test and the 2 mm cutoff test.

**Fig 5:**
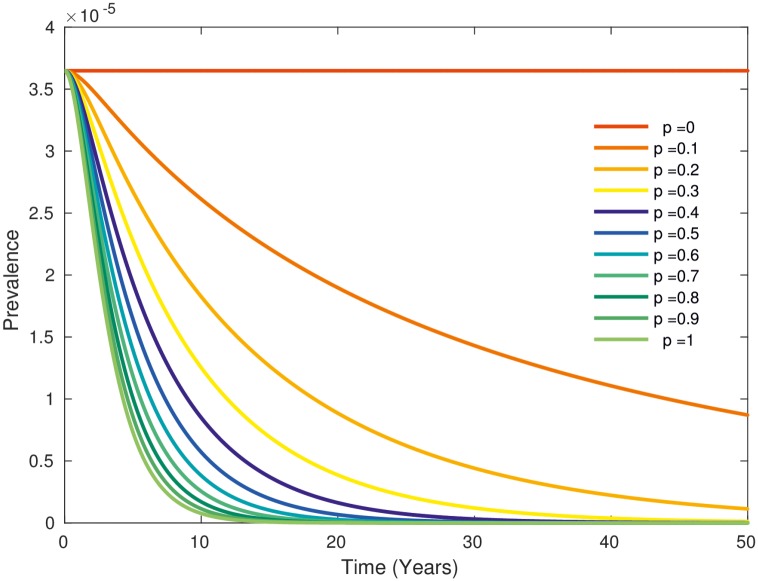
Relationship between human prevalence and proportion of test and slaughter using 4mm cut-off test.

The simulation of a test and slaughter intervention led to a decline in BTB prevalence depending on the proportion p of testing (Fig 3). The time to elimination, i.e. the time to reach an individual animal prevalence of less than one in a thousand, ranged from 75 years for p = 20% to 12 years for p = 100%. For values of p > 60%, the time to elimination was below 20 years (Fig 3). The reproductive number decreased rapidly below one with an increasing proportion test and slaughter p (Fig 4). With 60% testing and culling, the prevalence of exposed and active human BTB cases decreased from 3.5 per 1,000.000 to less than 1 in 1,000,000 at the time of freedom from disease after 20 years (Fig 5).

### Cost of test and slaughter intervention

**Fig 6:**
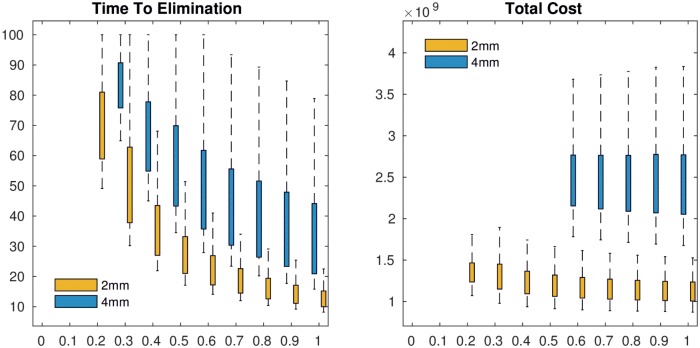
Total cost of the interventions that reach elimination for different proportions of tested animals and 2mm cut-off test (yellow) and 4mm cut off test (blue).

The cost of test and slaughter depends on the percentage p of test and slaughter (Table 5). Lower p results in lower cumulated costs but longer time until elimination. The cumulated cost is remarkably stable for p values higher than 0.2, ranging between 1.47 to 1.87 billion Euros within a time range of 12 to 75 years to reach freedom from disease. The cumulated cost of BTB test and slaughter intervention and the time to elimination were lower using 2mm cut-off of SICTT compared to the 4mm cut-off (Fig 6).

**Table 5:** Relationship of proportion of animals included in test and slaughter and the cumulated cost and time freedom from disease (Individual animal prevalence <1/1000).

Proportion of test and slaughter p	Cumulated cost of control in billions of euros* (4mm Test)	Time to reach freedom from disease in years (4mm Test)	Cumulated cost of control in billions of euros* (2mm Test)	Time to reach freedom from disease in years (2mm Test)
0		> 100		> 100
0.1		> 100		> 100
0.2		> 100	1.87	75
0.3		> 100	1.68	44
0.4		> 100	1.60	32
0.5		> 100	1.55	25
0.6		> 100	1.53	20
0.7	1.99	82	1.51	17
0.8	1.94	69	1.49	15
0.9	1.90	59	1.48	13
1	1.84	51	1.47	12

## Discussion and conclusions

This manuscript presents the first cattle to cattle and cattle to human compartmental deterministic mathematical model. Differential equations were used to describe different pathways within and between compartments at human and animal level. Sensitivity analysis of the model has been used to determine the most sensitive parameter. Additionally, different scenarios of test and culling interventions were simulated by considering different proportions of tested population per year (p).

## Model properties

To our knowledge this is the first model describing cattle to cattle and cattle to human transmission of BTB in Morocco, although an African buffalo-human model has been published [70]. Time series data similar to those for brucellosis in Mongolia [71] are unfortunately not available, but the available data allows for a parameterization under the assumption of endemic stability similar to Ethiopia [72]. The estimated reproductive number of 1.325 is in the range for both low risk areas (R_0_ = 0.6–1.4) and high risk areas (R_0_ = 1.3–1.9) reported for the United Kingdom [73] but is lower than the 1.7–2.2 reported by Bekara et al. for France [56]. Using a sensitivity analysis, the birth rate of cattle (b_c_) was determined to be the most sensitive parameter of the model.

A key challenge in this model was to distinguish between exposed and infected cattle because the diagnostic test utilized was the intradermal tuberculin test. We used the proportion of cattle with active TB (13.5%) among cattle tested positive by tuberculin skin test, as reported by Ngandolo et al [57], to calculate the number of infected cattle. Further microbiological data is required to better describe BTB prevalence in humans in Morocco. Patients treated for active BTB do not completely clear all organisms from their body, with some bacteria persisting in bone marrow [61]. Therefore, in contrast to our model, humans do not become completely susceptible again but technically are subject to re-infection [62]. We argue that this has only a minor impact on total human BTB prevalence, but re-infection should be considered to refine the model.

Our model contains many simplifications. The primary simplification is that of homogeneity: all cattle are not the same. Risk exposure of animals and humans to BTB could change according to sex and age, and we have ignored these differences. Contact between cattle is also not random and is far more likely within herds than between cattle in different herds. Many models have included this heterogeneity in contact patterns within and between herds [52–54], but we ignore it here because of a lack of data on within herd BTB transmission in Moroccan husbandry systems. Furthermore, although deterministic models provide reasonable estimates of mean behaviour in large populations, they cannot provide expected distributions of rare events. Therefore, they may not be appropriate for analysing very low transmission settings (that are necessary before elimination can occur). We circumvent this issue with a rather generous definition of elimination as prevalence of less than 1 in 1000.

### Test and slaughter intervention

BTB prevalence was found to reach less than one per thousand in less than 20 years when the proportion of tested cattle was above 60%. The annual cost for this potential intervention was nearly 77 million euros. Intervention in cattle was found to impact the prevalence of human TB due to *M*. *bovis*, which decreased from 5 per 10,000 to 1 per 10,000 after 17 years.

The economic assessment presented here is preliminary, and a detailed cost and cost-effectiveness analysis will be published separately. However, our analysis informs Moroccan bovine tuberculosis control policy on the time horizon, range of cost and optimal levels of intervention. An effective control program will depend on the human resources and technical and logistical capacity of the veterinary services to implement testing and slaughtering of animals. If the proportion of cattle subjected to test and slaughter was greater than 60%, freedom from disease would be reached in less than 20 years. The simulation results suggest that switching from a 4mm cut off to a 2mm cut off would be likely to result in significantly shorter durations of elimination programs and much cheaper elimination campaigns.

Our model simulates the removal of individual animals rather than whole herds. Past experience in Europe has shown that whole herd removal is critical for effective elimination in low prevalence situations [46]. In addition, a herd based model of the Moroccan cattle population could potentially lead to a lower intervention cost, as it is more realistic.

A recalculation of the intervention cost taking into account stratification by breed, sex and age should be undertaken, as it could lead to a different cost estimation of BTB control strategy.

Shortage of human resources should be considered for intervention planning, a maximum of 40% cull rate might be feasible; however, it would be costly in view of current Moroccan economic situation. One may think that test and slaughter implementation would lead to a reduction in cattle population and its by-product. But on the other hand, the increased import of cattle from other countries, with enhanced control measures, could maintain the current population density. In the meantime, as dairy products are provided mostly from highly controlled farms where BTB has a very low prevalence, we could argue that milk production would not be significantly affected.

### Towards one health

The WHO includes BTB amongst the seven neglected zoonoses which are perceived to be severe threats to public health [1]. Further molecular epidemiology investigations in Morocco are needed in order to clarify local and national human BTB/TB ratios. To reach this goal, closer collaboration, at the national and international level, between the human and animal health sectors through a One Health approach is highly recommended. Operations in these two sectors remain largely independent in Morocco. Communication must be enhanced to establish a One Health approach, which has proven efficacy in health service delivery and potential for economic savings in zoonosis control [74, 75].

## Supporting information

S1 Supporting information Calculation of cattle to cattle transmission rate. (PDF)

S2 Supporting information Calculation of cattle to human transmission rate. (PDF)

S3 Supporting information Calculation of birth rate. (XLSX)
