# Supplementary material for: Transmission dynamics and elimination potential of zoonotic tuberculosis in morocco
Source: PLoS Negl Trop Dis. 2017 Feb 2;11(2):e0005214. doi: 10.1371/journal.pntd.0005214 (PMC5289436; doi:10.1371/journal.pntd.0005214)
Supplement: S1 Supporting information — (PDF) [file pntd.0005214.s001.pdf]

Supporting Information 1: Calculation of the cattle to cattle transmission rate

The equations for the cattle population are given by

$$\frac{\mathrm{d}S(t)}{\mathrm{d}t} = bS(t) + r_b b(E(t) + I(t)) - \beta\frac{S(t)I(t)}{N(t)} - \mu S(t) - (1 - s_p)pS(t), \tag{1a}$$

$$\frac{\mathrm{d}E(t)}{\mathrm{d}t} = \beta\frac{S(t)I(t)}{N(t)} - \alpha E(t) - \mu E(t) - s_e pE(t), \tag{1b}$$

$$\frac{\mathrm{d}I(t)}{\mathrm{d}t} = \alpha E(t) - \mu I(t) - s_e pI(t), \tag{1c}$$

where $N(t) = S(t) + E(t) + I(t)$ and

$$\frac{\mathrm{d}N(t)}{\mathrm{d}t} = bS(t) + r_b b(E(t) + I(t)) - \mu N(t) - (1 - s_p)pS(t) - s_e p(E(t) + I(t)).$$

If $b > \mu$ and $p = 0$ the total cattle population, N, increases exponentially. In order to calculate the pre intervention endemic equilibrium we therefore define the proportion of susceptible, exposed and infected cattle as

$$s(t) := \frac{S(t)}{N(t)}, \quad e(t) := \frac{E(t)}{N(t)}, \quad i(t) := \frac{I(t)}{N(t)}.$$

Using the chain rule and equation (1b) we get

$$\frac{\mathrm{d}e(t)}{\mathrm{d}t} = (b - r_b)e(t)^2 - \beta i(t)^2 + (b - r_b - \beta)e(t)i(t) - (b + \alpha)e(t) + \beta i(t).$$

and from equation (1c)

$$\frac{\mathrm{d}i(t)}{\mathrm{d}t} = (b - r_b)i(t)^2 - (b - r_b)e(t)i(t) + \alpha e(t) - bi(t).$$

There exists an equilibrium proportion of exposed cattle, e_*, and infective cattle, i_*, such that

$$0 = (b - r_b)e_*^2 - \beta i_*^2 + (b - r_b - \beta)e_* i_* - (b + \alpha)e_* + \beta i_*.$$

and

$$0 = (b - r_b)i_*^2 - (b - r_b)e_* i_* + \alpha e_* - bi_*.$$

We now choose the transmission rate β such that

$$e_* + i_* = \phi,$$

where ϕ is the endemic prevalence before the intervention. This yields

$$i_* = \frac{\alpha\phi}{\alpha + b - (b - r_b b)\phi},$$

$$e_* = \phi - i_*$$

and

$$\beta = \frac{(b - r_b b)e_*^2 + (b - r_b b)e_* i_* - (b + \alpha)e_*}{i_*^2 + e_* i_* - i_*}.$$
