# Supplementary material for: Transmission dynamics and elimination potential of zoonotic tuberculosis in morocco
Source: PLoS Negl Trop Dis. 2017 Feb 2;11(2):e0005214. doi: 10.1371/journal.pntd.0005214 (PMC5289436; doi:10.1371/journal.pntd.0005214)
Supplement: S2 Supporting information — (PDF) [file pntd.0005214.s002.pdf]

Supporting Information 2: Calculation of the cattle to human transmission rate

The equations for the human population are given by

$$\frac{\mathrm{d}S(t)}{\mathrm{d}t} = bS(t) - \beta\frac{I_C(t)S(t)}{N(t)} - \mu S(t), \tag{1a}$$

$$\frac{\mathrm{d}E(t)}{\mathrm{d}t} = \beta\frac{I_C(t)S(t)}{N(t)} - \alpha E(t) - \mu E(t), \tag{1b}$$

$$\frac{\mathrm{d}I(t)}{\mathrm{d}t} = \alpha E(t) - \delta I(t) - \mu I(t), \tag{1c}$$

where $I_C(t)$ is the number of infected cattle at time t, $N(t) = S(t) + E(t) + I(t)$ and

$$\frac{\mathrm{d}N(t)}{\mathrm{d}t} = (b - \mu)N(t).$$

If $b > \mu$ the total human population, N, increases exponentially. In order to calculate the pre intervention endemic equilibrium we therefore define the proportion of susceptible, exposed and infected humans as

$$s(t) := \frac{S(t)}{N(t)}, \quad e(t) := \frac{E(t)}{N(t)}, \quad i(t) := \frac{I(t)}{N(t)}.$$

Using the chain rule and equation (1b) we get

$$\frac{\mathrm{d}e(t)}{\mathrm{d}t} = \beta i_C^* - \beta i_C^* e(t) - \beta i_C^* i(t) - \alpha e(t) - b e(t).$$

where i_C^* is the equilibrium proportion of infected cattle. Equation (1c) yields

$$\frac{\mathrm{d}i(t)}{\mathrm{d}t} = \alpha e - \delta i - b i.$$

There exists an equilibrium proportion of exposed humans, e_*, and infective humans, i_*, such that

$$0 = \beta i_C^* - \beta i_C^* e_* - \beta i_C^* i_* - \alpha e_* - b e_*.$$

and

$$0 = \alpha e_* - \delta i_* - b i_*.$$

We now choose the transmission rate β such that

$$e_* + i_* = \phi,$$

where ϕ is the endemic prevalence before the intervention. This yields

$$i_* = \frac{\alpha\phi}{\alpha + \delta + b},$$

$$e_* = \phi - i_*$$

and

$$\beta = \frac{(\alpha + b)e_*}{i_C^* s_*}.$$
